# Post Cholecystectomy Bile Duct Injury in an Acute Setting: Categorization, Triaging, and Management Algorithm

**DOI:** 10.7759/cureus.55828

**Published:** 2024-03-08

**Authors:** Divya Jain, Somanath Malage, Ashish Singh, Nalinikanta Ghosh, Rahul Rahul, Supriya Sharma, Ashok Kumar, Rajneesh K Singh, Anu Behari, Ashok Kumar, Rajan Saxena

**Affiliations:** 1 Surgical Gastroenterology, Sanjay Gandhi Postgraduate Institute of Medical Sciences, Lucknow, IND

**Keywords:** post cholecystectomy injury, management algorithm, biliary stricture, acute injury, bile duct injury

## Abstract

Background

Postcholecystectomy bile duct injury (BDI) is a management challenge with significant morbidity, mortality, and effects on long-term quality of life. Early referral to a specialized hepatobiliary center and appropriate early management are crucial to improving outcomes and overall quality of life. In this retrospective analysis, we examined patients who were managed at our center over the past 10 years and proposed a triage and management algorithm for BDI in acute settings.

Methods

Patients referred to our center with BDI from January 2011 to December 2020 were reviewed retrospectively. The primary objective of initial management is to control sepsis and minimize BDI-related morbidity and mortality. All the patients were resuscitated with intravenous fluid, antibiotics (preferably culture-based), correction of electrolyte deficiencies, and organ support if required. A triage module and management algorithm were framed based on our experience. All the patients were triaged based on the presence or absence of bile leaks. Each group was further subdivided into red, yellow, and green zones (depending on the presence of sepsis, organ failure, and associated injuries), and the results were analyzed as per the proposed algorithm.

Results

One hundred twenty-eight patients with acute BDI were referred to us during the study period, and 116 patients had BDI with a bile leak and 12 patients were without a bile leak. Out of bile leak patients, 106 patients (91.38%) had sepsis with or without organ failure (red and yellow zone) and required invasive intervention in the form of PCD insertion (n=99, 85.34%) and/or laparotomy, lavage, and drainage (n=7, 6.03%). Another 10 patients (8.62%) had controlled external biliary fistula (green zone), of which four were managed with antibiotics, four underwent endoscopic retrograde cholangiopancreatography stenting, and only two (1.7%) patients could undergo Roux-en-Y hepaticojejunostomy upfront due to late referral. Among patients with BDI without bile leaks, nine (75%) had cholangitis (red and yellow zones). Out of these, five required PTBD along with antibiotics and four were managed with antibiotics alone. Only three (25%) patients in this group could undergo definitive repair without any restriction on the timing of referral and were sepsis-free at presentation (green zone). A total of nine patients had a vascular injury, and four of them required digital subtraction angiography and coil embolization. There were three (2.34%) mortalities; all were in the red zone of rest and had successful initial management. In total, five patients were managed with early repair in the acute setting, and the rest underwent definitive intervention at subsequent admissions after being converted to green zone patients with initial management.

Conclusion

The presented categorization, triaging, and management algorithm provides optimum insight to understand the severity, simplify these complex scenarios, expedite the decision-making process, and thus enhance patient outcomes in early acute settings following BDI.

## Introduction

Cholecystectomy is considered a "gold standard" procedure for gallstone disease [1] and represents one of the most common surgeries performed by surgeons worldwide. Although various guidelines have been proposed to prevent bile duct injury (BDI) [2-4], the incidence of BDI has remained consistent at 0.1-0.5% over the past three decades [5], with subsequent high morbidity and mortality (2-4%) [6]. Unfortunately, most of the patients are referred late, often having undergone multiple interventions and having received suboptimal medical care. This can be attributed, in part, to medicolegal concerns related to iatrogenic injury and to a lack of understanding of the disease.

Early referral to a specialized hepatobiliary center and appropriate early management are crucial to reduce morbidity and mortality and to improve overall quality of life [7]. In this retrospective analysis, we examined 128 patients who were managed at our center over the past 10 years. Based on our findings, we proposed a triage and management algorithm for BDI in acute settings.

## Materials and methods

A retrospective analysis was conducted on a prospective database of patients with acute BDI who had been referred to us from January 2011 to December 2020. We proposed a color-coded triage and management algorithm based on our experience in managing these patients. We analyzed our 10-year retrospective data results after putting them into the algorithm.

Initial management, grouping, and color coding

The primary objective of initial management is to control sepsis and minimize BDI-related morbidity and mortality. All the patients were resuscitated with intravenous fluid; antibiotics (preferably culture-based), correction of electrolyte abnormalities, and organ support were provided if required. A detailed pre-operative, intra-operative, and post-operative history and examination were obtained to assess the mode of injury, site of injury, and clinical evidence of sepsis. Subsequently, a hemogram, liver function test, kidney function test, and blood gas analysis were obtained to identify metabolic derangements. Abdominal ultrasonography (USG) was done in all the patients with acute BDI to look for liver (biliary radical dilatation, abscess), extrahepatic biliary tract (if feasible), intra-abdominal collections, and pleural effusion.

All acute BDI cases were classified into two groups: Group I (BDI with a bile leak) and Group II (BDI without a bile leak). Group I consisted of patients with controlled or uncontrolled external biliary fistula (EBF), bilioma, and biliary peritonitis. Controlled or uncontrolled biliary fistulas are defined on the basis of the absence or presence of undrained intraperitoneal collections, respectively. Bilioma is localized bile collection outside the biliary tree, while biliary peritonitis is considered when bile is present in the whole of the abdominal cavity and is associated with clinical signs of peritonitis. Group II consisted of patients who presented with jaundice resulting from bile duct ligation or clipping without any evidence of a bile leak.

**Figure 1:**
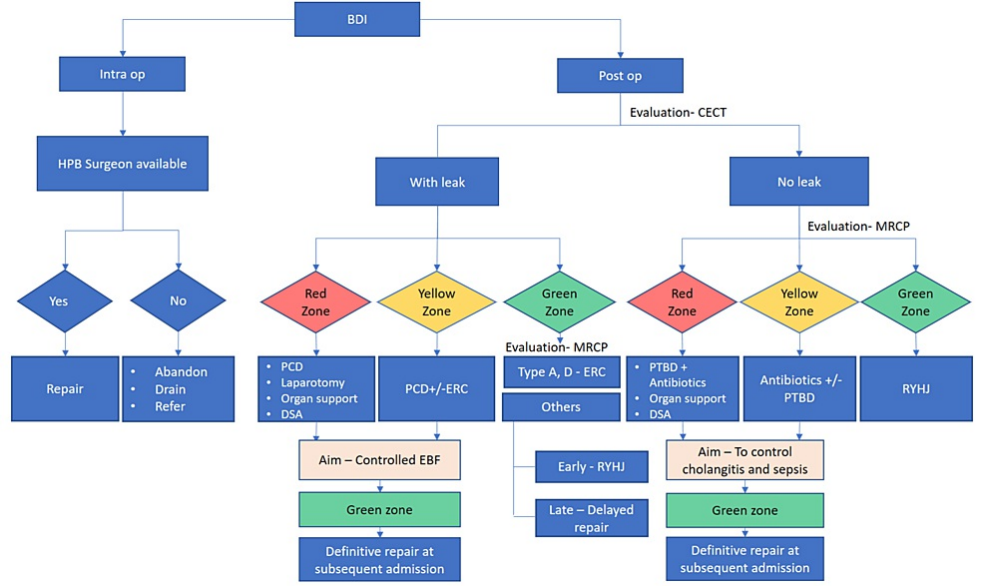
Management algorithm PCD: percutaneous drainage, DSA: digital subtraction angiography, ERC: endoscopic retrograde cholangiopancreatography, RYHJ: Roux-en-Y hepaticojejunostomy, EBF: external biliary fistula, MRCP: magnetic resonance cholangiopancreatography, CECT: contrast-enhanced computed tomography, BDI: bile duct injury

Subsequently, patients from both groups were further color-coded based on the presence of sepsis, organ failure, and associated injuries into red, yellow, and green zones, as depicted in Table 1. Patients classified under the red group requiring organ support were managed in the intensive care unit (ICU), while patients in the yellow group were managed in the high-dependency unit (HDU). Patients categorized under the green group were managed in the general ward. A management algorithm was proposed based on the triage group, as depicted in Figure 1.

**Table 1:** Parameters for triage and triage criteria ICU: intensive care unit, HDU: high-dependency unit, *only for Group I patients with bile leak

Clinical characteristics	Red zone	Yellow zone	Green zone
Intra-abdominal collection*	Present	Present	Absent
Cholangitis/sepsis	present	Present	Absent
Organ failure	Present	Absent	Absent
Associated injury	Present/absent	Absent	Absent
Managed at	ICU	HDU	Ward

Further evaluation and management

A computed tomography (CT) scan of the lower thorax, abdomen, and pelvis with or without intravenous contrast was performed, depending on their renal parameters and contrast allergy, to identify intra-abdominal collections, liver abscess, pleural effusion, and a gross estimation of biliary anatomy. Also, associated vascular and visceral injuries can be assessed at the same time. Depending on USG and CT scan findings, these patients were grouped as patients with or without bile leaks and further triaged as per the protocol (Table 1).

Group I

Red and yellow zone patients were managed using a step-up approach, starting with percutaneous catheter drainage (PCD) and progressing to laparotomy if required to establish a controlled EBF. Magnetic resonance cholangiopancreatography (MRCP) was done in green zone patients to delineate biliary anatomy and to select possible definitive management options in the same setting, such as endoscopic retrograde cholangiopancreatography (ERC) and stenting (for Strasburg A or D injuries) or definitive surgery, i.e., Roux-en-Y hepaticojejunostomy (RYHJ), if the patient presented early (within seven days).

Group II

All patients were further evaluated with MRCP to identify the level of biliary block. Patients in the red and yellow groups were managed with antibiotics, with or without percutaneous transhepatic biliary drainage (PTBD), to address cholangitis. Patients in the green group underwent RYHJ in the same setting, irrespective of the duration of referral.

Associated injury

Patients suspected to have a vascular injury clinically (anemia, hemorrhagic drain output, requirement of blood transfusion) or biochemically (fall in hemoglobin) or surgeon-reported vascular injury underwent a triple-phase CT scan. Patients with evidence of active contrast extravasation or pseudoaneurysm were managed with digital subtraction angiography (DSA) and embolization or stenting as appropriate. Patients with duodenal and colonic injuries were evaluated with CT enterography using oral contrast and managed as per the algorithm depicted in Figure 1.

For patients in whom definitive treatment could not be offered in the acute setting, primary management was aimed at shifting them from the red or yellow zone to the green zone and planning delayed definitive repair at the subsequent admission after biliary mapping is done using MRCP. The success of this approach in an acute setting was determined by the ability to discharge patients without sepsis.

## Results

Patient cohort

A total of 128 patients presented with acute BDI. The median age of our cohort was 37 years (range 17-79 years), and the majority were females (73.4%). The mean duration of referral from the day of cholecystectomy was 25 days, with a range of one to 57 days. Before referral, 69 (54%) patients had already undergone multiple radiological investigations and interventions, including laparotomies. The details of the demographic profile and other characteristics of patients are shown in Table 2.

**Table 2:** Clinical profile of the cohort PCD: percutaneous drainage, ERC: endoscopic retrograde cholangiopancreatography

Parameters	No. of patients N (%)
Male	34 (26.6%)
Female	94 (73.4%)
Pre-operative symptoms	
Recurrent biliary colic	108 (84.4%)
Acute calculus cholecystitis	11(8.6%)
Cholangitis	6 (4.7%)
Asymptomatic cholelithiasis	3 (2.3%)
Index surgery	
Laparoscopic cholecystectomy	57 (44.5%)
Lap converted to open cholecystectomy	16 (12.5%)
Open cholecystectomy	55 (43%)
Elective surgery	125 (97.7%)
Emergency surgery	3 (2.3%)
Interventions before referral	
Aspiration	18 (14%)
PCD	16 (12.5%)
ERC	15 (11.7%)
Laparotomy and lavage +/- attempted repair	20 (15.6%)

Presentation

Out of a total cohort of 128 patients, 116 (90.6%) presented with bile leak (Group I), while 12 patients (9.4%) did not have bile leak (Group II). Within Group I, only 10 (8.6%) patients presented with controlled EBF (green zone). The rest were in the red or yellow zone, with 66 having uncontrolled bile leaks, six presenting with biliary peritonitis, and 34 patients having bilioma. In Group II, nine (75%) patients presented with cholangitis, and only three (25%) patients were without sepsis (green zone). Nine patients had an associated vascular injury, two had a duodenal injury, and one had a colonic injury.

Management of patients of group I (bile leak)

**Figure 2:**
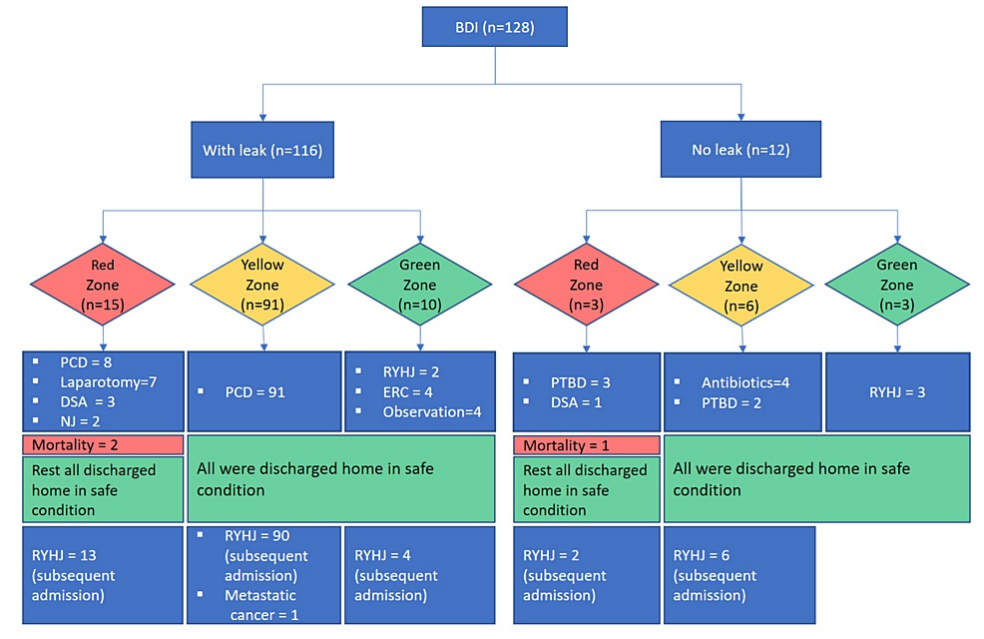
Patient cohort managed according to the algorithm BDI: bile duct injury, PCD: percutaneous drainage, DSA: digital subtraction angiography, NJ: nasojejunal tube, RYHJ: Roux-en-Y hepaticojejunostomy, ERC: endoscopic retrograde cholangiopancreatography, PTBD: percutaneous transhepatic biliary drainage

Among the 116 patients with bile leak, only 10 (8.7%) were sepsis-free (green zone), while the remaining 106 (91.3%) had intra-abdominal collections and sepsis, with or without organ failure (red and yellow zone) (Figure 2).

Red Zone

Eight (53.3%) patients were managed with PCD insertion, while seven (46.6%) subsequently required exploratory laparotomy due to persistent sepsis or peritonitis. The goal of surgical exploration in these patients was to do lavage and drainage of all the collections to make a controlled EBF. Two patients in this zone had mortality due to septic shock.

Yellow Zone

All patients (n=91) included in this group were managed with PCD insertion and culture-based antibiotics.

Green Zone

Out of 10 patients in the green zone, ERC stenting was done for four patients (three for type A injury and one for type D injury); two patients with early presentation (within seven days) underwent RYHJ on post-operative day (POD) one and sixth of index surgery. Four patients who presented late underwent billion-dollar reconstructive surgery at subsequent admissions.

Management of patients of group II (without bile leak)

Red Zone

All patients (n=3) were managed with PTBD and culture-based antibiotics. One of these patients had an associated vascular injury, underwent DSA and embolization of the right hepatic artery, but unfortunately could not survive (mentioned subsequently) (Figure 2).

Yellow Zone

All patients (n=6) in this zone were managed with antibiotics. However, two patients required PTBD subsequently.

Green Zone

Three patients with good nutrition status and no sepsis underwent RYHJ on POD 8, 15, and 35 following index surgery.

Patients with associated injuries

Among nine patients with associated vascular injuries, four had right hepatic artery (RHA) pseudoaneurysms that were managed with DSA and coiling. The remaining five patients did not show any contrast extravasation or pseudoaneurysm on CT angiography and were managed conservatively. Out of these, two patients had ligated RHA, one patient had right portal vein thrombosis with atrophy of the right lobe, and two patients had right anterior sectoral perfusion defects. Two patients with duodenal injury were managed with nasojejunal tube (NJ) placement, while one patient with colonic injury required a diversion stoma.

Outcome

All patients were managed according to the principles described above. Except for three (2.3%) deaths, all were discharged in sepsis-free conditions. Out of five patients who underwent definitive RYHJ, four (80%) had McDonald A, and one had McDonald D [8] at 36 months of follow-up. The remaining 120 patients were discharged home in sepsis-free conditions with or without a controlled EBF. Out of them, four patients were managed with ERC and stenting, and 115 underwent definitive repair (RYHJ) on subsequent admission after biliary mapping with MRCP before surgery, once they belonged to the green zone. The results of delayed biliary repair were similar to the previously published data from our institute (more than 90% McDonald A and B) [9]. One patient was found to have malignancy on histopathological evaluation of slides and subsequently developed metastatic disease, so definitive surgery could not be performed. The median (IQR) duration of hospital stay at index admission was 11 (10) days (range 4-40 days).

Mortality

All three patients who had mortality were referred late and were classified in the red zone (two in Group I, one in Group II). First, presented on POD 12 with bilio-sanguinous drain output, peritonitis, sepsis, and acute lung injury, underwent exploratory laparotomy with lavage and drainage for biliary peritonitis on the same day. Her sepsis could not improve after surgery; rather, organ failure worsened, and he died six days after surgery. Second, she was referred to us on POD 32 with intra-abdominal collection, cholangitis, sepsis, and acute kidney injury. PCD and PTBD insertions were done along with ICU care and organ support. However, she subsequently developed multiple organ dysfunction syndrome (MODS) and died due to cardiac arrest on day 35 of admission. The third (in Group II) had an associated vascular injury (blunt vertebral artery injury) and presented with disseminated intravascular coagulation, septic shock, and MODS on POD 13. Unfortunately, the patient died within 48 hours of admission.

## Discussion

BDI can be fatal in acute settings and can result in life-threatening complications like cholangitis, cholangitic abscesses, bleeding or secondary biliary cirrhosis, and portal hypertension in the long term if managed inadequately [10]. So, initial management is important to achieve a good long-term outcome that could vary from antibiotics with or without PCD or PTBD insertion to definitive billion-enteric anastomosis along with the management of associated injuries.

While the literature suggests an increased risk of BDI in emergency settings for acute cholecystitis (0.58% vs. 0.18%) [11], elective cholecystectomy done for biliary colic or asymptomatic gallbladder stones makes up the largest cohort of BDI (86.7% in our study), since it is the most common indication of cholecystectomy. Thus, it emphasizes the importance of safe cholecystectomy even in uncomplicated gallstone disease [12,13].

If a BDI is detected during cholecystectomy, on-table repair by a hepatobiliary surgeon may reduce short-term morbidity [14]. This also results in shorter hospital stays, lower hospitalization costs, and favorable long-term outcomes [15,16]. In our study, most patients were referred late, with a mean time of referral of 25 days and a maximum of 57 days following index surgery.

As the majority of cholecystectomies are performed by non-hepatobiliary surgeons at less-equipped peripheral centers, early repair is challenging. Previous unsuccessful repair attempts not only complicate subsequent repairs but also have adverse long-term outcomes [17,18]. Thus, all these patients should be referred to a specialist hepatobiliary center as early as possible. In our study, 20 patients underwent laparotomy before referral, and four of them attempted unsuccessful repair before referral. The primary objective of managing BDI in an acute setting is not to map the biliary anatomy or provide definitive repair but rather to effectively control sepsis and save a patient's life [19]. A very selected population of patients with BDI in an acute setting (falling in the green zone of triage) can be managed with definitive intervention in the index admission. Most of the patients do require management of sepsis, and definitive intervention is considered only after control of sepsis and adequate pre-habilitation at subsequent admission. Early referral not only reduces morbidity and mortality, but it also provides the opportunity for definitive intervention in suitable patients to reduce subsequent readmissions and long-term painful and cumbersome stays with PCDs and/or PTBDs. Only five patients (4.03%) could undergo early repair in our cohort. Four (80%) of them had good outcomes at 40 months of follow-up.

Our study shows that the BDI with a bile leak and the BDI without a bile leak are two separate sets of patients with different investigative and management protocols. It also demonstrates that the color coding stratifies the patients based on the severity and complexity of the disease, as the mortalities were only in the red zone patients. On progressing from the green to the red zone, interventions required were also escalated.

Also, the algorithm identifies indications of radiological investigation, with a CT scan abdomen being the investigation of choice to look for intra-abdominal sources of sepsis and associated injury [20,21], while MRCP is required for biliary mapping when definitive intervention is planned [22,23]. Thus, avoiding unnecessary investigations will improve the outcome and reduce the financial, social, and emotional burden on families. Similarly, ERC is recommended only when biliary-enteric continuity is maintained, as in type A and type D injuries. Fifteen patients in our study underwent ERC before referral, even though it was not indicated.

To the best of our knowledge, this is the first study to address the management of BDI in the initial acute setting, which provides a categorization, color-coding triage based on the gravity of the disease, and management algorithm to ease the decision-making of the clinicians with a clear road map. This study offers an appropriate categorization, triage, and systematic stepwise management algorithm that should be considered as a vital supplement to clinical judgment in the decision-making process of managing BDI in acute settings. The limitations of this study include the retrospective nature of the study, which is associated with selection bias. The algorithm was based on the management experience of such patients, which needs prospective validation.

## Conclusions

The management of patients with acute BDI injuries is complex and requires a multidisciplinary team (including an interventional radiologist, a gastroenterologist, and an experienced hepatobiliary surgeon) to curtail morbidity and mortality. Surgeons encountering BDI may not be well equipped and experienced enough to manage such patients; hence, early referral to specialized hepatobiliary centers is a must. The presented categorization, triaging, and management algorithm provides optimum insight to understand the severity, simplify these complex scenarios, expedite the decision-making process, and thus enhance patient outcomes. Further studies are needed to substantiate the usefulness of this algorithmic approach to the management of acute BDI.
